# Summer Recreations

**Published:** 1869-09

**Authors:** 


### SUMMER RECREATIONS.

Many have gone to the sea-shore, or to watering-places, to get more vigorous health and better appetites. Neither in summer or winter, do we want a more comfortable or enjoyable place than Gotham, anywhere in the neighborhood of Union and Madison squares. One of the most desirable localities is Irving Place, the coolest, cleanest, and perhaps one of the most quiet streets in the city, half as broad again as Chestnut street, of Philadelphia, only six blocks long, headed by Gramercy Park above, with Fourteenth street and the Academy of Music at the other end, within one block of Union Square and two blocks of Stuyvesant Park, with the sea-breezes from the broad Atlantic, a few miles to the eastward, and not a shop or store, but one, in its whole length, lined with residences on either side, generally owned by those who occupy them—we verily believe that people who cannot make themselves comfortable there, even in the hottest summer, must have dyspepsia, and could not be made comfortable anywhere. Twelve cents will purchase a ride of two or three miles and back, north or south, and twelve cents more a jaunt to Greenwood and back, or a trip down the Bay of six or eight miles, to Staten Island or the Narrows; and more than these, the opportunity, any day or hour, of a walk, dry-shod, of as many miles as may be desired; whereas, in the winter, a walk in the country is impracticable. In the summer-time, we admit that an early walk under green trees, and beside the running streams, with the happy twitterings of innumerable birds, is delightful to think of; but the realization is not delightful at all, for the dews of the grass and the dust of the road return the pedestrian with boots so soiled, and skirts so draggled, that the operation is not repeated in a season. If we wait until the dew has dried from the grass, the sun beams down so insufferably hot, that we are not tempted from the house at all; and from eleven until three, we literally pant and swelter, even at Far Rockaway.

To gain health and large mental profit at the same time, we advise all who can, to ride on horseback, in companies of two or three of congenial minds, and travel directly West, to the Missouri, or to Kansas, on the other side, in easy stages of thirty-three miles a day, lying by for three or four hours in the middle of the day, making short excursions on foot, or conversing with the neighbors about the climate, soil, productions, and character of the locality, and its inhabitants; returning by a route rather different, either striking across to the North-east, from Independence, or St. Joseph's, up to Superior City, in Minnesota, at the south-western extremity of Lake Superior, destined by the way, we think, to be a second Chicago; or deflecting to the South, strike on Memphis, thence across Tennessee, through the beautiful City of the Cedars, Nashville, thence across the mountains on the great turnpike, to Chattanooga and Knoxville—taking care to call on that most amiable, accomplished artist in ivory miniature, JAMES W. DODGE, who, in his mountain-home, in White county, near Claysville, spends his summer in the cultivation of fruits and flowers, the taste for which is so nearly allied to that of painting. At Chattanooga, go to Table Rock, overlooking the town, said to be one of the most exquisitely beautiful and picturesque localities in the Old World or the New. Thence go to the Springs of Virginia, and onward, home. Such a journey, entered upon with resolution, as to its effects on the mind and body, present and future, as laying the foundation for useful reflections in after years, to young men and old, sick or well, and to unmarried women, is utterly beyond comparison with the lazy, loafing, heating, gourmandizing, swilling, gambling life, which is more or less to be met with at all public places of general summer resort.

To persons who have more means and time, or higher ambitions, we say: Go to Liverpool or Southampton, in some of our ocean steamers, from fifty to a hundred and thirty dollars, according to the accommodations. Steamers to Southampton charge eighty dollars for first-cabin fare, and fifty for second cabin. Put all your baggage in a common hand-valise, as we did, and snap your fingers at all custom-house officers, porters, hackmen, and robbers; go first to Bangor, in Wales, to the slate-quarries, Menai Bridge, &c., *on foot!* Thence to Dublin, and branch out in any direction; after seeing the ordinary sights, then turn your attention to the extraordinary ones, the most instructive, and such as never come under the notice of the common herd of excursionists—we mean, the peat-bogs, the farms, the mud and stone houses by the wayside, go in and talk with the inmates, make yourself one of them, eat of their potatoes, sleep on their straw beds, and ask and answer all questions. In this way you will know something of the country and people you have visited, and will obtain subjects for remark, and illustration, and amusement, for yourself and children, and friends, for the remainder of a lifetime. If you have plenty of time to spare, go north, to the linen factories of famed Belfast, to the Giant's Causeway, and across to the home of Burns, to Glasgow, and stately Edinburgh; then make for London, *via* Abbottsford, Berwick-upon-Tweed, but save London for another season, if possible. It will take a whole year to map out London, and beautiful Paris another. Still, all may be done by two or three energetic young men in company, during the five or six months of summer, at an expense of less than six hundred dollars each. We did it ourselves, and could do it the second time, for less than three hundred. A more delightful time we never spent: such an excitement, such joyousness, such vigor, such an appetite, and such sleep—such a glorious time we never had before, a younger brother along; unless it was when, some years before, we explored a wilderness in the Western wilds, and in six months, never slept under a roof, or ate at a civilized table—sleeping on the ground; the sky, a canopy; the Indian, our hucksters. But as it is so easy to go down, we would not advise any unfledged youth to try the experiment of travelling among, and associating with savages. Our own country is the most picturesque and grand in the world; and that the summers of our youth, and men and women of leisure and means, and of our invalids, are spent in the enervating debaucheries of fashionable places of resort, instead of explorations such as we have named, is discreditable to our intelligence as a people, and is a degradation to our morals and physical nature, as individuals.

If there is not force of character enough to take these jaunts, or time, or means, or health, do not allow, then go to some retired country place, from ten to a hundred miles away from all steamboat and railway lines, where board can be had for two dollars a week, and the daily use of a horse for several hours, at a still lower price, and spend every moment of that time on his back, with regular and rational habits of eating, drinking, and sleeping—taking also a liberal amount of manual and pedestrian exercises, in addition; then, and then only, may pleasure-seekers reasonably expect to return to the city with bodies and minds re-invigorated for the pursuits of business. But we have not failed, in some years, to notice, that various persons who had returned from their summer jaunts became suddenly ill and died: and such will be the history of others this season, simply because, on their return, they will eat with the appetite which was begotten by the constant excitement of travel and exercise in the open air, while they are engaged in the old routine of business, with not one-tenth part of the exhilarating exercises so recently taken; and thus it is, that summer recreations result in but little permanent good. The remedy is, to be satisfied, at least for a month after returning to town, with two meals a day, eating nothing between times, and nothing later than four o'clock in the afternoon, with an hour or two's walk, or ride on horseback, or other exhilarating exercise in the open air, every day, rain or shine.

